# Is a central cavity necessary for bioactive glass-ceramic spacers in plated ACDF? A retrospective comparison of solid versus cavity designs

**DOI:** 10.1007/s00402-026-06389-y

**Published:** 2026-06-18

**Authors:** Tae Hoon Kang, Geumho Lee, Byungjun Kang, Jeongwoon Han, Minjoon Cho, Jae Hyup Lee

**Affiliations:** 1 Department of Orthopedic Surgery, College of Medicine, Seoul National University, Seoul, Korea, Republic of; 2 Department of Orthopedic Surgery, SMG-SNU BRM Medical Center, Seoul, Korea, Republic of; 3 Department of Orthopedic Surgery, Daniel Medical Center, Bucheon, Korea, Republic of; 4 Department of Orthopedic Surgery, Plus Orthopedic hospital, Incheon, Korea, Republic of; 5 Department of Orthopedic surgery, Sungae hospital, Seoul, Korea, Republic of

**Keywords:** Anterior cervical discectomy and fusion, Spinal fusion, Interbody cage, Prosthesis design, Biocompatible materials, Bioactive glass-ceramic

## Abstract

**Purpose:**

Traditionally, interbody cages feature a central cavity for graft packing. However, for bioactive glass-ceramic (BGS-7) spacers achieving fusion via surface-mediated osseointegration, this cavity may be biologically redundant and structurally disadvantageous. We compared radiological and clinical outcomes of plated anterior cervical discectomy and fusion (ACDF) using BGS-7 spacers with or without a central cavity.

**Methods:**

We retrospectively reviewed patients undergoing plated ACDF using solid non-cavity (17 patients, 24 levels) or central-cavity (17 patients, 33 levels) BGS-7 spacers. Radiographic fusion was evaluated using dynamic interspinous distance (primary, ≤ 2 mm) and segmental angular motion (secondary, ≤ 4°), alongside CT-based osseointegration (direct contact ≥ 50%). Subsidence (≥ 2 mm) and clinical outcomes were evaluated at 12 months.

**Results:**

Both groups demonstrated comparable 12-month clinical improvements (*p* > 0.05). Radiographic fusion rates were equivalent between non-cavity and central-cavity groups based on the primary distance (91.7% vs. 84.8%, *p* = 0.687) and secondary angular criteria (79.2% vs. 63.6%, *p* = 0.331). The non-cavity group showed a trend toward superior segmental stability, with a smaller dynamic gap distance (0.82 ± 0.64 mm vs. 1.53 ± 1.99 mm, *p* = 0.064). CT-based fusion perfectly matched (90.5% vs. 90.9%, *p* = 1.000). Subsidence rates were comparably low (12.5% vs. 12.1%), with no spacer breakage.

**Conclusions:**

In plated ACDF, adding a central cavity to BGS-7 spacers confers no measurable advantage over the solid design. Solid spacers inherently preserve maximum endplate contact area for optimal load-sharing and surface-mediated fusion. Eliminating the cavity and supplemental grafts avoids logical redundancy, donor-site morbidity, and unnecessary healthcare costs.

## Introduction

Anterior cervical discectomy and fusion (ACDF) is a well-established surgical technique widely used for the treatment of degenerative cervical spine disorders, demonstrating excellent clinical and radiologic outcomes [23]. Traditionally, autologous bone grafts, particularly those harvested from the iliac crest, have been considered the gold standard for achieving interbody fusion. However, donor-site complications such as pain and infection, as well as limited graft quantity, have prompted the increasing use of alternative materials including allografts and synthetic spacers [4]. 

Among these, bioactive glass-ceramic (BGS) composed of a CaO–SiO₂–P₂O₅–B₂O₃ system, known as BGS-7, has attracted attention due to its excellent osteoconductivity, biodegradability, and biocompatibility. Both experimental and clinical studies have demonstrated that BGS-7 cages achieve fusion rates comparable to or exceeding those of titanium cages, hydroxyapatite spacers, and autologous bone grafts, suggesting that it is a promising alternative biomaterial [12–14, 19].

One of the BGS-7 spacers, NOVOMAX Fusion (CG Bio, Seoul, Republic of Korea), was originally developed as a non-cavity spacer with its geometry optimized by finite element modeling (FEM) to provide superior mechanical strength [3, 8–10, 22] Unlike conventional interbody cages that rely on a macroporous central cavity for three-dimensional trabecular bone ingrowth, BGS-7 achieves fusion primarily through surface-mediated osteoconduction. Upon implantation, it forms an apatite-like layer on its surface, enabling direct chemical bonding at the spacer–endplate interface [12, 16] Despite this innate surface-bonding mechanism, a modified version featuring a central cavity was later introduced by the manufacturer, presumably intended to allow for the packing of bone graft materials. Notably, this design modification was implemented without prior clinical or biomechanical evidence demonstrating explicit superiority over the original non-cavity design. Furthermore, this design reduces the load-bearing cross-sectional area, thereby compromising mechanical strength and increasing the risk of cage subsidence or structural failure [24].

To date, no study has directly compared the radiologic and clinical outcomes of BGS-7 spacers with and without a central cavity in ACDF procedures. Evaluating the impact of these structural differences on fusion rate, mechanical stability, and clinical recovery is essential for optimal implant selection and surgical decision-making. Therefore, the primary objective of this retrospective study was to compare plated ACDF using non-cavity versus central-cavity BGS-7 spacers, ultimately to determine whether this design modification provides any measurable clinical benefit.

## Materials and methods

### Ethics statement

We conducted this study in compliance with the principles of the Declaration of Helsinki. The study protocol was reviewed and approved by the Institutional Review Board of [Blinded] (IRB No. 40-2025-45). Owing to the retrospective nature of the study and the use of anonymized data, the requirement for informed consent was waived.

### Study design and patients

This was a retrospective single-center comparative study. Patients who underwent ACDF using BGS-7 spacers between January 2014 and December 2022 were identified from the operative log at [Blinded] All the operations were performed by a single senior surgeon [Blinded] who had more than 10 years experiences of ACDF.

Inclusion criteria were as follows: (1) age ≥ 18 years; (2) ACDF using BGS-7 spacers at one or more cervical levels for radiculopathy or myelopathy; and (3) availability of clinical and radiologic follow-up of at least 12 months. Exclusion criteria were: (1) combined posterior fusion or anterior cervical corpectomy and fusion; (2) history of previous cervical spine surgery; (3) inadequate X-ray follow-up at the 12-month postoperative time point; and (4) incomplete clinical records.

Patients were categorized into two groups according to the spacer design used at the time of surgery: non-cavity BGS-7 spacers (non-cavity group) versus central-cavity BGS-7 spacers (cavity group). As the manufacturer modified the spacer design during the study period, the patients essentially formed sequential cohorts. Therefore, this study represents a retrospective observational comparison between patients treated before and after this design transition.

### Surgical procedure

All patients underwent routine ACDF via a standard anterior Smith-Robinson approach. Following conventional discectomy, adequate neural decompression, and careful endplate preparation, an appropriately sized BGS-7 spacer was inserted, and the segment was stabilized with an anterior cervical plate and screws [3]. 

The primary technical difference between the two groups pertained to the application of supplemental graft materials. When a central-cavity spacer was used, the central hole was densely packed with a mixture of local autologous bone and additional bone substitutes, such as demineralized bone matrix (DBM). Any remaining graft material was also spread around the spacer. Conversely, in the non-cavity group, the BGS-7 spacer was inserted into the disc space without the use of any additional bone grafts or substitutes. A closed-suction drain was routinely placed and was typically removed within 1 to 2 days postoperatively once the drainage amount had sufficiently decreased.

### Spacer design

Both the non-cavity and central-cavity BGS-7 spacers shared an identical rectangular footprint measuring 13 mm in width and 15 mm in depth (total footprint area of 195 mm²), and were available in various heights to accommodate individual patient anatomy.

**Fig. 1:**
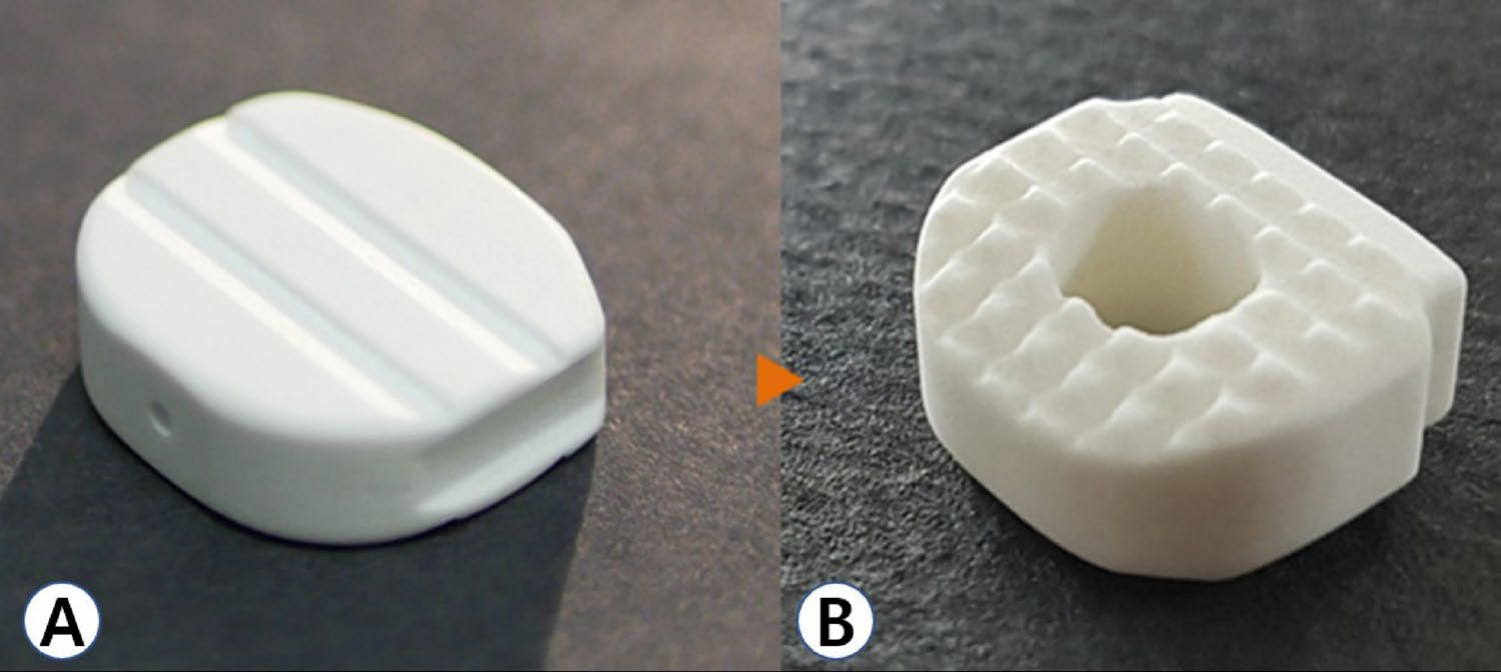
Photographs illustrating the two bioactive glass-ceramic (BGS-7) spacer designs evaluated in this study. Both designs share an identical 13 × 15 mm rectangular footprint (total area: 195 mm²). **A** The original non-cavity spacer features a solid structure with longitudinal groove, preserving the maximal endplate-contacting surface. **B** The modified central-cavity spacer incorporates a 4-mm-diameter central hole intended for bone graft packing, which reduces the effective contact area by approximately 12.6 mm² (6.4%). It also features a pyramidal textured pattern on the articulating surfaces

The original non-cavity spacer (Fig. 1A) featured a solid ceramic block with a slightly convex superior surface containing longitudinal grooves to conform to the vertebral endplates, preserving the full 195 mm² effective contact area. In contrast, the modified central-cavity spacer (Fig. 1B) incorporated a 4-mm-diameter cylindrical hole in the center. This cavity inherently reduced the effective surface contact area by approximately 12.6 mm² (a 6.4% reduction, yielding 182.4 mm²) compared to the solid design. Furthermore, the articulating surfaces of the central-cavity spacer were altered to include a pyramidal textured pattern (teeth) intended to increase initial friction against the endplates.

### Radiological evaluation

**Fig. 2:**
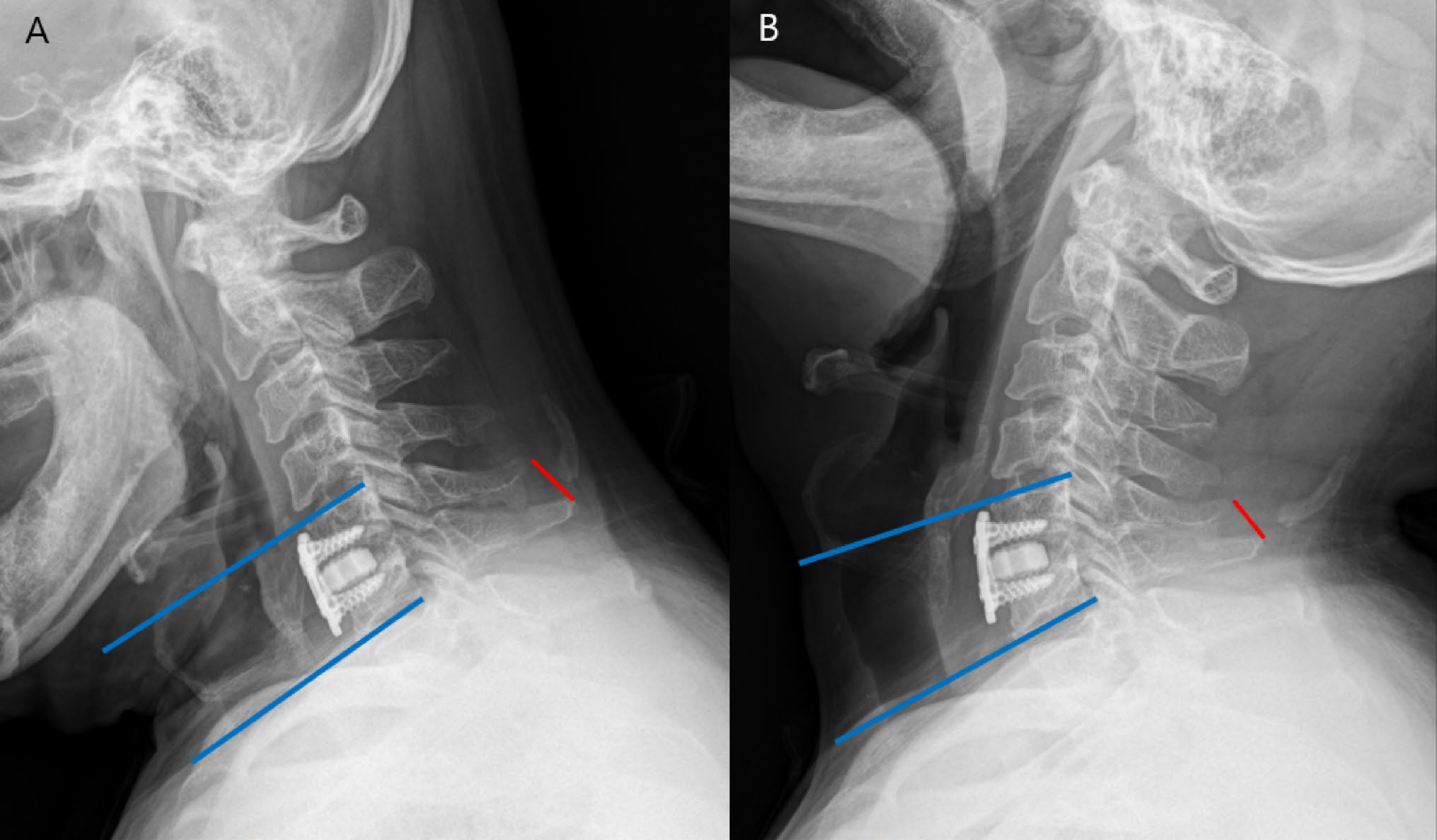
Dynamic lateral radiographs used to evaluate radiographic fusion based on segmental stability. **A** Flexion and **B** extension views. The red lines indicate the measurement of the interspinous distance, while the blue lines illustrate the measurement of the segmental angle at the fused level. Radiographic fusion is strictly defined as an interspinous distance difference of ≤ 2 mm and a segmental angular motion of ≤ 4° between flexion and extension. Movement exceeding either threshold is considered indicative of radiographic pseudarthrosis

Radiographic fusion was evaluated based on segmental stability using dynamic flexion–extension radiographs. Following established radiologic criteria, segmental stability was assessed using two independent parameters: interspinous distance and segmental angular motion. Because interspinous distance change (≤ 2 mm) is one of the most widely accepted and reproducible indicators for evaluating pseudarthrosis, we utilized it as the primary criterion for dynamic radiographic fusion. Additionally, a segmental angular motion of ≤ 4° was evaluated as a secondary supporting metric [5, 21] (Fig. 2).

Furthermore, 12-month CT scans were analyzed using sagittal and coronal reconstructions. Because BGS-7 spacers without cavity are radiopaque and bond directly to bone via a surface apatite layer, CT-based fusion cannot be evaluated by conventional internal trabecular bridging through a central cavity. Currently, universally established CT criteria for evaluating solid bioactive spacers are lacking. Therefore, we adopted a recently described methodology to quantitatively assess the extent of direct osseointegration at the spacer–endplate interfaces [21]. On the representative mid-sagittal and mid-coronal cuts, the degree of fusion was calculated as the ratio of the continuous bone-bridging line (the absence of a radiolucent gap) to the total length of the cage interface. In this study, successful CT fusion was defined as demonstrating direct bone-to-spacer contact involving ≥ 50% of the interface at both the superior and inferior vertebral endplates.

### Assessment of spacer subsidence and breakage

Spacer subsidence was defined as a ≥ 2 mm loss of interbody height relative to the immediate postoperative lateral radiograph, measured at the anterior and posterior disc space at 6 and 12 months postoperatively. Spacer breakage was defined as the appearance of a new radiolucent line or gap within the spacer body on 12-month radiographs, suggesting structural failure [21]. 

### Measurement reliability

To minimize measurement bias, all radiographic variables were separately evaluated by two independent spine surgeons [Blinded] who were blinded to both the clinical data and the spacer design. Interobserver reliability for continuous measurements was assessed using the intraclass correlation coefficient (ICC) with a two-way random-effects model and absolute agreement definition. In this study, the ICC values for all key radiographic parameters were ≥ 0.80, indicating good to excellent interobserver reliability. Any discrepancies between the observers were resolved by joint review and consensus, which was used for the final analysis.

### Clinical outcomes assessment

Baseline clinical assessments included the Japanese Orthopaedic Association (JOA) score, Neck Disability Index (NDI), Functional Rating Index (FRI), and visual analog scale (VAS) scores for neck, trapezial, and radicular pain. These clinical parameters were longitudinally evaluated at approximately 6 and 12 months postoperatively.

### Statistical analysis

Statistical analyses were performed using R software (version 3.6.3; R Foundation for Statistical Computing, Vienna, Austria). Continuous variables are presented as mean ± standard deviation, and categorical data as frequencies and percentages. Group comparisons were conducted using the independent t-test or Mann-Whitney U-test for continuous variables, and the chi-square or Fisher’s exact test for categorical variables. Within-group longitudinal changes were evaluated using paired t-tests or Wilcoxon signed-rank tests. A *p*-value < 0.05 was considered statistically significant.

## Results

### Patient demographics and baseline characteristics

A total of 34 patients (57 fused levels) met the inclusion criteria: 17 patients (24 levels) in the non-cavity group and 17 patients (33 levels) in the central-cavity group. Baseline demographic and clinical characteristics—including age, sex distribution, proportion of smokers, bone mineral density (DEXA T-scores), preoperative disc height, and spacer height—were comparable between the two groups, with no significant differences (all *p* > 0.05). Detailed demographic data and operative details are summarized in Table 1.

**Table 1:** Demographic & radiographic data

Patient (*N* = 34)	Non-cavity (*n* = 17)	Central cavity (*n* = 17)	*p*-value
Age	61.59 ± 10.86	58.53 ± 10.20	0.404 t
Sex(male/female)	12/5 (70.59%/29.41%)	10/7 (58.8%/41.2%)	0.473 †
Smokers	7 (41.2%)	2 (11.8%)	0.118 ‡
DEXA T-score	-0.98 ± 1.07	-0.81 ± 1.23	0.669 t
Osteoporosis	2 (11.8%)	2 (11.8%)	1.000 ‡
Disease type			1.000‡
Central stenosis	14 (82.4%)	15 (88.2%)	
Foraminal stenosis	3 (17.6%)	2 (11.8%)	
Surgery level			0.286 ‡
1 level	1 (5.9%)	5 (29.4%)	
2 level	10 (58.8%)	8 (47.1%)	
3 level	5 (29.4%)	4 (23.5%)	
4 level	1 (5.9%)	0 (0%)	

Levels (*N* = 57)	*N* = 24	*N* = 33	*p*-value
Operation level			0.212 ‡
C3-4	3 (12.50%)	3 (9.09%)	
C4-5	11 (45.83%)	7 (21.21%)	
C5-6	6 (25.00%)	13 (39.39%)	
C6-7	2 (8.33%)	8 (24.24%)	
C7-T1	2 (8.33%)	2 (6.06%)	
Pre-operation disc height	3.97 ± 0.76	4.23 ± 0.78	0.211 t
Spacer height			0.191 ‡
5 mm	3 (12.50%)	0 (0.00%)	
6 mm	3 (12.50%)	8 (24.24%)	
7 mm	11 (45.83%)	14 (42.42%)	
8 mm	7 (29.17%)	11 (33.33%)	

Data presented as mean ± standard deviation; *t* T-test; † Chi-square test; ‡ Fisher’s exact test, *DEXA* dual energy X-ray absorptiometry

### Radiologic outcomes

Both groups demonstrated a marked reduction in flexion–extension segmental motion postoperatively. At 12 months, the mean dynamic interspinous gap distance was 0.82 ± 0.64 mm in the non-cavity group and 1.53 ± 1.99 mm in the central-cavity group (*p* = 0.064). Similarly, the mean angular motion was 2.32 ± 1.79° in the non-cavity group and 3.13 ± 2.20° in the central-cavity group (*p* = 0.133).

Based on the primary criterion of an interspinous distance difference of ≤ 2 mm, the 12-month dynamic radiographic fusion rate was 91.7% (22/24 levels) in the non-cavity group and 84.8% (28/33 levels) in the central-cavity group (*p* = 0.687). Furthermore, when evaluated by the secondary criterion of angular motion (≤ 4°), acceptable stability was observed in 79.2% (19/24 levels) and 63.6% (21/33 levels) of the levels, respectively (*p* = 0.331).

At the 12-month CT follow-up, successful osseous fusion was achieved in 90.5% (19/21 levels) of the non-cavity group and 90.9% (30/33 levels) of the central-cavity group (*p* = 1.000).

Regarding complications, spacer subsidence (≥ 2 mm) occurred at comparably low rates: 12.5% (3/24 levels) in the non-cavity group and 12.1% (4/33 levels) in the central-cavity group. Importantly, no cases of spacer breakage or structural failure were detected in either group throughout the follow-up period. (Table 2).

**Table 2:** Radiologic outcomes comparing non-cavity and central-cavity BGS-7 spacers

	Non-cavity (*n* = 24 levels)	Central cavity (*n* = 33 levels)	*p*-value
Dynamic segmental motion			
6 months Postoperatively			
Interspinous gap distance (mm)	1.04 ± 0.82	1.70 ± 2.02	0.096 t
Angular motion (°)	2.53 ± 1.29	3.13 ± 2.00	0.177 †
12 months Postoperatively			
Interspinous gap distance (mm)	0.82 ± 0.64	1.53 ± 1.99	0.064 t
Angular motion (°)	2.32 ± 1.79	3.13 ± 2.20	0.133 †
Radiographic fusion rate at 6 months			
Primary criterion (distance ≤ 2 mm)	83.3% (20/24)	78.8% (26/33)	0.745 ‡
Secondary criterion (angle ≤ 4°)	79.2% (19/24)	66.7% (22/33)	0.460 ‡
Radiographic fusion rate at 12 months			
Primary criterion (distance ≤ 2 mm)	91.7% (22/24)	84.9% (28/33)	0.687 ‡
Secondary criterion (angle ≤ 4°)	79.2% (19/24)	63.6% (21/33)	0.331 ‡
CT fusion (continuous bone-bridging ≥ 50%)	90.5% (19/21)	90.9% (30/33)	1.000 ‡
Complications			
Spacer subsidence (≥ 2 mm)	12.5% (3/24)	12.1% (4/33)	> 0.999
Spacer breakage	0% (0/24)	0% (0/33)	N/A

Data are presented as mean ± standard deviation or percentage (number/total levels)

*N/A* not applicable

†Analyzed using the independent t-test or Mann-Whitney U-test

‡Analyzed using the chi-square test or Fisher’s exact test CT scans at 12 months were available for 21 levels in the non-cavity group and 33 levels in the central-cavity group

### Clinical outcomes

All clinical parameters significantly improved from baseline to 12 months postoperatively in both groups. However, there were no significant between-group differences in the JOA score, NDI, FRI, or VAS scores for neck, trapezial, and radicular pain at the 12-month follow-up (all *p* > 0.05) (Table 3).

**Table 3:** Clinical outcomes

Outcome measure	Non-cavity (*n* = 17)	Central-cavity (*n* = 17)	*p*-value*
JOA score			
Preoperative	12.24 ± 2.60	10.56 ± 3.29	0.109
12 months postop	14.53 ± 2.53	14.56 ± 2.76	0.974
Improvement (Δ)	2.29 ± 1.17	4.00 ± 4.42	0.141
NDI			
Preoperative	16.71 ± 5.27	20.41 ± 7.40	0.102
12 months postop	9.41 ± 2.45	11.12 ± 3.43	0.105
Improvement (Δ)	7.29 ± 4.16	9.29 ± 7.29	0.335
FRI			
Preoperative	20.76 ± 6.09	24.00 ± 4.95	0.099
12 months postop	12.18 ± 3.15	13.41 ± 3.43	0.282
Improvement (Δ)	8.59 ± 5.36	10.59 ± 4.17	0.234
VAS neck pain			
Preoperative	3.35 ± 2.15	3.29 ± 1.61	0.929
12 months postop	0.65 ± 0.86	1.00 ± 1.22	0.339
Improvement (Δ)	2.71 ± 1.83	2.29 ± 1.72	0.504
VAS trapezial pain			
Preoperative	3.53 ± 2.53	4.41 ± 2.37	0.302
12 months postop	0.71 ± 0.92	1.18 ± 1.29	0.23
Improvement (Δ)	2.82 ± 2.43	3.24 ± 2.63	0.639
VAS radicular pain			
Preoperative	4.29 ± 2.52	5.06 ± 1.89	0.324
12 months postop	0.94 ± 0.75	1.06 ± 1.34	0.755
Improvement (Δ)	3.35 ± 2.42	4.00 ± 1.97	0.399

Data presented as mean ± standard deviation, *JOA* Japanese Orthopaedic Association, *NDI* Neck Disability Index, *FRI* Functional Rating Index, *VAS*: visual analog scales, *t* T-test

## Discussion

In this retrospective comparative study, we investigated whether the addition of a central cavity to BGS-7 spacers—and the routine packing of this cavity with supplemental graft material—confers any clinical or radiologic advantage over the original non-cavity design in plated ACDF. The most notable finding was that, despite the theoretical rationale that a central cavity might facilitate bone bridging, we observed no measurable benefit in terms of primary distance-based radiographic fusion rates, CT-based continuous osseointegration, or 12-month clinical outcomes. Furthermore, both designs maintained excellent structural integrity, with comparable rates of subsidence and no instances of spacer breakage. These results suggest that the inherent surface-bonding properties of BGS-7 may be sufficient for achieving successful fusion in ACDF, rendering the structural modification of a central cavity and the subsequent need for additional bone grafting redundant in this clinical context.

Our results, demonstrating a dynamic radiographic fusion rate of over 90% (based on the primary distance criterion) and a CT-based fusion rate of approximately 90% in both groups, are consistent with previous reports on BGS-7 and other established interbody devices. This underscores the reliability of BGS-7 in achieving robust osseointegration at the spacer–endplate interface [3, 7].

Traditionally, the central cavity of an interbody cage is designed as a sanctuary for bone grafts, facilitating three-dimensional bone ingrowth and bridging—a necessary process for bioinert materials like PEEK or metal [2]. In contrast, BGS-7 is a bioactive glass-ceramic that undergoes a surface ion-exchange process in the physiological environment, leading to the formation of a bone-like apatite layer [10–13, 15, 17, 18] This layer enables direct chemical bonding with the vertebral endplates, a phenomenon known as surface-mediated osteoconduction. Because fusion with BGS-7 is predominantly driven by this expansive surface integration rather than by localized bridging through a macroporous architecture, the structural requirement for a central cavity may be inherently less critical. Our findings suggest that the solid surface of the non-cavity design provides an ample substrate for this bioactive bonding, achieving high-quality fusion without the biological necessity of an internal graft chamber.

Regarding segmental stability, we employed a rigorous dual-criteria evaluation. Consistent with previous systematic reviews highlighting interspinous distance as a highly reproducible and widely adopted indicator of fusion, we utilized a translational threshold (≤ 2 mm) as our primary determinant [21]. Under this standard, both the solid and cavity-bearing spacers achieved excellent and comparable fusion rates (91.7% vs. 84.8%). Furthermore, when applying the secondary angular motion threshold of ≤ 4°, the non-cavity group demonstrated a robust fusion rate of 79.2%, compared to 63.6% in the central-cavity group. Although the differences in categorical fusion rates did not reach statistical significance, the continuous variables revealed a compelling biomechanical trend. The non-cavity design consistently exhibited superior segmental stability, highlighted by a noticeably smaller dynamic gap distance at 12 months (0.82 mm vs. 1.53 mm, *p* = 0.064).

We attribute the lack of a statistically significant difference between the two designs to two primary factors. First, the routine use of an anterior plate-screw construct provides robust immediate and rigid stabilization, which likely overshadows any minor biomechanical differences originating from the interbody spacer design itself [1]. Consequently, while both designs achieved comparable fusion in our plated cohort, the structural disadvantage of a cavity design—specifically the reduced contact area—could potentially compromise both segmental stability and the ultimate fusion rate in a stand-alone ACDF setting where interbody mechanics play a more critical role. Second, the residual motion at the fused segments was inherently minute—mostly restricted to under 1 to 2 mm—creating a ‘floor effect.’ Combined with our relatively small sample size, detecting a definitive statistical divergence within such a narrow range of motion becomes exceedingly difficult. Ultimately, these dynamic motion results indicate that adding a central cavity provides no additional biomechanical advantage in a plated ACDF setting.

Beyond dynamic motion, the solid non-cavity BGS-7 spacer offers distinct structural advantages. By avoiding the approximately 6.4% footprint reduction caused by a 4-mm central cavity, the solid design (195 mm² total footprint) optimizes axial load distribution and maximizes the surface area available for apatite layer formation and direct osseointegration [27]. 

Theoretically, the reduced contact area of the cavity design could increase stress concentration, predisposing the segment to subsidence or implant breakage. However, both groups demonstrated comparably low subsidence rates (~ 12%) and zero breakages. This is likely because the routine anterior plate-screw construct neutralizes the biomechanical disadvantages of a reduced footprint. Crucially, in a stand-alone ACDF setting lacking such rigid supplemental fixation, this structural vulnerability would likely become far more pronounced, significantly increasing the risk of severe subsidence and implant failure [1]. Additionally, subclinical micro-subsidence (< 2 mm) may have remained undetected [27]. 

Finally, evaluating CT-based fusion for bioactive spacers remains challenging. The microscopic apatite layer responsible for surface-mediated osseointegration is largely sub-radiographic on conventional 1-mm CT slices [3, 13, 19]. Furthermore, extrapolating a few 2D linear approximations to a full 3D footprint introduces spatial sampling bias, highlighting the need for future 3D volumetric interface analyses to more accurately quantify true surface-bonding fusion.

Beyond radiological outcomes, the routine use of a central cavity introduces substantial clinical and economic burdens. Cavity designs mandate supplemental grafting for internal bridging. However, iliac crest autografts carry known donor-site morbidities, and local osteophytes often provide insufficient volume [26]. Consequently, surgeons must resort to costly alternatives like DBM or peptides, or rhBMP-2, which carries well-documented risks of catastrophic soft-tissue swelling in the cervical spine [20, 25]. Furthermore, packing a bioactive ceramic cavity with other synthetic ceramics (e.g., bioglass) presents a logical redundancy [19]. In the current era of value-based spine care, minimizing unnecessary healthcare expenditures without compromising clinical efficacy is of critical importance [6]. Because adding a cavity and graft material offers no measurable clinical benefit over the solid BGS-7 design, the associated operative time, potential morbidity, and healthcare costs are ultimately unjustified.

This study has several limitations. First, it is a retrospective analysis with a relatively small sample size. However, this sample size was inherently and unavoidably constrained because the manufacturer completely discontinued the original non-cavity BGS-7 spacer during the study period. Second, this sequential shift in implant availability introduced an unavoidable temporal bias, making it physically impossible to accrue additional non-cavity cases, match surgical dates, or conduct a prospective randomized controlled trial (RCT). Third, owing to the retrospective nature of the cohort, complete 12-month CT follow-up was not perfectly captured for all operated levels, slightly limiting the radiological evaluation. Given that prospective clinical comparisons (e.g., RCTs) are no longer feasible due to the discontinuation of the solid design, future investigations should focus on in silico finite element (FE) analyses. Such biomechanical studies are necessary to directly compare the stress distribution and safety margins against cage breakage between the solid and cavity-bearing BGS-7 spacers under extreme physiological loading conditions.

## Conclusion

In plated ACDF, the structural modification of adding a central cavity to bioactive glass-ceramic (BGS-7) spacers confers no measurable biological, biomechanical, or clinical advantage over the original solid non-cavity design. Both configurations achieve comparably high fusion rates, excellent clinical recovery, and low rates of subsidence. Given that the solid spacer inherently preserves a maximized effective contact area for optimal load-sharing and provides a broader canvas for surface-mediated osseointegration, the routine addition of a central cavity and the subsequent need for supplemental bone grafts are biologically redundant and economically unjustified. Therefore, the simpler, solid non-cavity BGS-7 spacer represents a highly effective, structurally sound, and cost-conscious choice for plated ACDF.

## Data Availability

The datasets generated and/or analyzed during the current study are not publicly available due to patient privacy but are available from the corresponding author on reasonable request.
